# The developmental expression dynamics of *Drosophila melanogaster *transcription factors

**DOI:** 10.1186/gb-2010-11-4-r40

**Published:** 2010-04-12

**Authors:** Boris Adryan, Sarah A Teichmann

**Affiliations:** 1 Computational Biology Group, Structural Studies Division, MRC Laboratory of Molecular Biology, Hills Road, Cambridge CB2 2QH, UK; 2 Cambridge Systems Biology Centre and Department of Genetics, University of Cambridge, Downing Street, Cambridge CB2 3EH, UK

## Abstract

**Background:**

Site-specific transcription factors (TFs) are coordinators of developmental and physiological gene expression programs. Their binding to *cis*-regulatory modules of target genes mediates the precise cell- and context-specific activation and repression of genes. The expression of TFs should therefore reflect the core expression program of each cell.

**Results:**

We studied the expression dynamics of about 750 TFs using the available genomics resources in *Drosophila melanogaster*. We find that 95% of these TFs are expressed at some point during embryonic development, with a peak roughly between 10 and 12 hours after egg laying, the core stages of organogenesis. We address the differential utilization of DNA-binding domains in different developmental programs systematically in a spatio-temporal context, and show that the zinc finger class of TFs is predominantly early expressed, while Homeobox TFs exhibit later expression in embryogenesis.

**Conclusions:**

Previous work, dissecting *cis*-regulatory modules during *Drosophila *development, suggests that TFs are deployed in groups acting in a cooperative manner. In contrast, we find that there is rapid exchange of co-expressed partners amongst the fly TFs, at rates similar to the genome-wide dynamics of co-expression clusters. This suggests there may also be a high level of combinatorial complexity of TFs at *cis*-regulatory modules.

## Abstract 2

High levels of combinatorial complexity of transcription factors during embryogenesis in Drosophila melanogaster shed light on mechanisms of transcriptional control.

## Background

In the post-genomic era, transcriptional regulatory relationships are often displayed in the form of a gene regulatory network (GRN), in which the nodes represent genes and the edges are the regulatory interaction of 'activation' or 'repression'. Developmental geneticists turned to 'network biology' decades ago, because the complex relationships between genes can often best be summarized in a wiring diagram. A good example is the early patterning of the *Drosophila *embryo, a relatively well understood process involving the concerted action of about ten or so transcription factors (TFs). The anterior-posterior axis of the fly embryo is initially defined by a maternally deployed protein gradient of the Bicoid (Bcd) TF. Regionalized gap gene expression domains establish a genetic circuitry that defines pair rule and segment polarity gene expression (see [1] for review). This GRN model allows one to make inferences about the behavior of the network upon perturbation (for example, in a logical analysis [2] or in an experimental approach [3]). These works clearly show the added value of a systems approach. Note that signal transduction cascades are not disregarded in these models; they provide the connections between otherwise separate GRNs. However, within specific developmental lineages, the body plans of animals primarily underlie the concerted action of TFs within GRNs (see [4,5] for review).

GRNs are hierarchical structures that can be dissected into functional modules and network motifs (see [6] for review). Single input motifs in which a target gene receives regulatory input from only one TF are commonly seen in unicellular organisms [7]. Most genes involved in metazoan development, however, require more complex modes of regulation: multiple TFs contribute to the expression state of the target gene. At the heart of this regulation lie the *cis*-regulatory modules, genomic clusters of specific binding sites for TFs (see [8] for review). The availability of TFs to bind to those *cis*-regulatory modules is fine-tuned and plays an important role in the precise regulation of the genes. One of the most intriguing examples for the modulation of developmental expression is the 'stripe 2' enhancer of the gene *even skipped *(*eve*) in *Drosophila*. This pair rule gene is expressed in seven stripes along the anterior-posterior axis of the early embryo. Individual stripes or combinations of stripes are under the control of individual enhancers. The possibly best-characterized enhancer drives the expression of the second stripe [9,10]. This enhancer contains activating binding sites for the TFs Bcd and Hunchback (Hb), and repressing binding sites for Giant (Gt) and Krüppel (Kr). At one, and only one position within the fly embryo (a domain of only a few nuclei in width), the gradients of the maternal TFs and the regionalized gap genes establish an environment under which the 'stripe 2' enhancer can drive expression of *eve*.

The microenvironment under which gene expression through a *cis*-regulatory module is facilitated is thus primarily, but not exclusively, influenced by the qualitative and quantitative presence of binding TFs [11]. The structures of the factors themselves provide additional means to modulate network function [12]. Some TF classes such as the zinc finger TFs recognize their target sequences as monomers, while other classes often require binding as homo- or heterodimers, at foremost the developmentally important helix-loop-helix (HLH) TFs [13]. This has direct implications for the minimal TF configuration necessary to drive transcription. Together, the availability of a TF in a given biological context as well as the combination of the TFs modulate the biological outcome.

For the life cycle of unicellular organisms such as yeast, a large proportion of the TFs and their regulatory interconnections have been identified [14]. Even the dynamics of the entire GRN under varying physiological conditions has been elucidated [15,16]. Metazoan development is clearly more complex, and although there has been progress in the description of some developmental GRNs at a systems level in model organisms [17-19] and many individual studies, we lack fundamental information about the number, structure and functions of their regulatory networks. For most organisms we even lack reliable details about the TFs contributing to the development of tissues and organ systems. And for those organisms and tissues where we have this information, we at most know a few of their interconnections.

As the first step in the analysis of GRNs, Bolouri and Davidson [20] propose a strategy to obtain 'a parts list' and then 'map how these parts fit together'. We have recently undertaken an investigation to create such a parts list and have defined the repertoire of site-specific TFs in *Drosophila melanogaster*, work that resulted in the FlyTF database [21]. Experimental studies have determined the genome-wide binding of some of these TFs (see, for example, [22]). A comprehensive screen aiming to determine the binding of the majority of factors is underway as part of the modENCODE projects [23]. We have started a computational analysis of currently available genomics data in *Drosophila *in order to shed light on developmental TFs and their role in GRNs.

Here, we present our results to integrate the repertoire of *D. melanogaster *TFs with information about their expression. We determined that approximately 95% of the roughly 750 TFs are utilized in a developmental context. For those TFs where spatio-temporal gene expression information is available, we investigated which tissues showed the highest degree of TF involvement. Interestingly, we found that the embryonic nervous system does not only express more genes than other body parts, but also expresses a high percentage of TFs compared to other body parts. There is considerable overlap between the TF expression repertoires of tissues, such that only a small fraction of TFs exhibits real tissue-specificity. Interestingly, tissue-specificity is not strictly conserved between embryonic and adult tissues, though we do observe nervous system-specific expression of TFs conserved between the two stages. For the first time in a metazoan organism, we show the degree of TF exchange between developmental co-expression clusters, which we interpret as an approximation of their mode of collaboration in different tissues.

## Results and discussion

### DNA-binding domain families in the *Drosophila *transcription factor repertoire

Our previous work using computational predictions and a large-scale literature curation identified 753 site-specific TFs in *D. melanogaster *[21]. Most can uniquely be mapped to 731 genes (97% of the repertoire), whereas 22 are only assigned to a transcription-related or DNA-binding 'activity' that has not been assigned to any gene. An example for the latter group is the Glue enhancer binding factor-I (FBgn0013970) [24].

Fifty different types of DNA-binding domains (DBDs) are encoded in the fly genome. Our previous work established that only 14 types of DBDs are present in more than 5 TFs (Additional file 1). The zinc finger DBD of the C2H2 type is the most abundant domain and can be found in 249 TFs, which is also the case in most other eukaryotic genomes [25,26]. The Homeobox is the second most common DBD with 99 counts. Other frequently used DBDs are the helix-loop-helix (55 counts), zf-C4 type zinc finger (22 counts), the BESS (20 counts) and the Forkhead domains (19 counts), and 19 TFs with various bZIP-like leucine zipper DBDs. In contrast, there are single copy DBDs in well-established TFs that occur only once or twice in the *Drosophila *genome - for example, the SRF (Mef2, Blistered), the Stat (Stat92E) and the Prox1 (Prospero) domains.

A compilation of all raw data used in this study is available as 'mini-website' in Additional file 2.

### Almost all transcription factors are expressed in embryonic development

Assuming that gene expression implies the activity of the encoded protein, we first assessed TF activity in development and physiology. Independent of the experimental strategy used for data acquisition, the proportion of TFs active at some stage during embryonic development is high. Active transcription [27] overlapping with at least part of the gene can be observed for 95% of TFs (694 of 731 TFs on the array). In the developmental time-course experiment published by Hooper *et al*. [28], 98% of TFs (667 of the 679 TFs present on the array) show either average or high expression during embryogenesis. This is in keeping with the results obtained by *in situ *hybridization [29,30], by which 94% of TFs (351 of 373 TFs in the database) are detected in the embryo.

We were also interested in the utilization of TFs in adult tissues as their gene expression profiles should exhibit the footprint of both morphological and physiological homeostasis. About 94% of the site-specific TFs (687 of 724 TFs present on the array) are expressed in one or more adult tissues in the FlyAtlas [31].

### Four broad classes of TF expression in embryonic development

**Figure 1:**
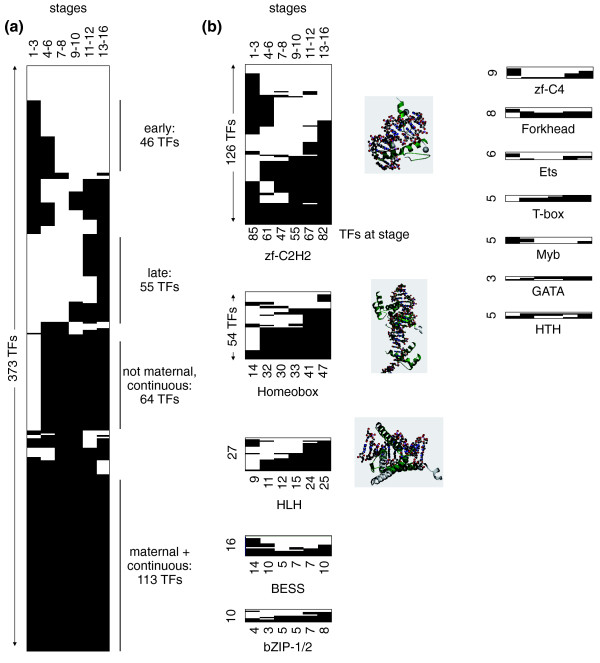
**Patterns of temporal utilization of site-specific transcription factors**. **(a) **Binary representation of TF expression according to the BDGP *in situ *database: black, expressed; white, not expressed. Expression behavior can be roughly categorized into six classes, no embryonic expression, diverse expression, early TFs, late TFs, those with continuous zygotic expression and continuous expression (including maternal contribution). The four largest classes are detailed in the main text. **(b) **Temporal utilization for different TF families. Absolute numbers of expressed TFs are provided underneath each panel. Note that roughly half of the TFs per family have been covered by the BDGP. HTH: helix-turn-helix.

Figure 1a shows a binary matrix of embryonic TF expression based on the Berkeley *Drosophila *Genome Project (BDGP) *in situ *database. From the matrix, there are four broad expression patterns amongst the TFs: (a) early expression only (46 TFs); (b) late expression only (55 TFs); (c) not maternal but continuous expression (64 TFs); (d) and maternal and continuous expression (113 TFs).

We were especially intrigued by the large number of TFs that are maternally contributed (those of classes a and d): 58.7% (219 of 373) of the TFs expressed during stages 1 to 3 in the BDGP database. This clearly shows that the maternal contribution is not restricted just to the dozen or so TFs that participate in axis determination, but may be involved in developmental processes beyond.

In the microarray experiments, we interpreted gene expression based on embryo material younger than 2 h after egg lay (AEL) as maternal contribution. In the time-course published by Hooper *et al*. [28], 48.2% (327 of 679) of TFs show high expression at 1 to 2 h AEL. Although by 2 h AEL a number of genes are expressed from the zygotic genome, this compares to 47.1% (337 of 715) of TFs in the 0 to 30 minute AEL data point of [32], and significant early expression is further supported by the 58.7% (429 of 731) of expressed TFs in the 0 to 2 h AEL data point in the active transcription map. Although the thresholds for the microarray experiments are somewhat arbitrarily chosen, there is general agreement and consistency between the different approaches of gene expression profiling.

We restricted our further analysis to the BDGP dataset as each gene is manually curated and spatially resolved [29,30]. A significant proportion, roughly one-third, of the maternally contributed factors is expressed in neuronal structures at later developmental stages (please refer to Additional file 3 for details.)

In many other publications, individual duplicate TFs are dissected in terms of their conservation or divergence in expression patterns [13,33-37]. Here, we survey global trends of expression for large families of distantly related TFs that share a DBD. We observe a tendency for members of DBD TF families to have broadly similar timing of expression during embryogenesis (Figure 1b; Additional file 4). For instance, the number of TFs in the Homeobox and HLH families that are expressed during embryogenesis increases (Figure 1b). In contrast, the zf-C2H2 family includes 82 TFs expressed in the last stage, but also 85 members that are present in the earliest stage. Notably, almost half of these early TFs belong to the subfamily of zf-C2H2 TFs that also contain a zinc finger-associated domain (zf-AD), while many fewer of the late TFs have this additional domain.

Viewed as a percentage of all TFs present at each stage, the zinc finger family is strongly represented at the earliest stage (Additional file 4). Of the 46 exclusively early TFs in the BDGP database, half utilize a zinc finger DBD (15% zinc finger TFs, and 35% zinc finger with zf-AD TFs). In contrast, the number of HLH TFs (0% to 15%) and Homeobox TFs (4% to 27%) starts low and then increases significantly throughout embryogenesis (χ^2 ^test, *P *< 0.001). The trends observed for the *in situ *hybridization-based approach are confirmed on a genome-wide level in the microarray studies (Additional file 4).

The microarray data cover a longer time span of embryogenesis than the BDGP and so extend our view: they show that the percentage of factors with a zinc finger out of all expressed TFs decreases continuously until the very end of embryogenesis, while the percentage of Homeobox TFs remains high. At the same time, beyond the temporal coverage of the BDGP database, the bZIP class of TFs increases its share of all expressed TFs between 16 and 24 h AEL in the Hooper *et al*. dataset. This is consistent with their biological function, as more than a quarter of the bZIP TFs have annotations connected to late embryonic or larval development, with an over-representation of Gene Ontology (GO) term 'instar larval or pupal development' reflecting this (*P *< 0.002). Microarray data further support the continuous expression of about half of the zf-C4 hormone receptor TFs, an important finding considering that, for example, an essential role for ecdysone signaling in embryogenesis has only been established relatively recently [38].

The functional subdivision of TFs and their expression behavior suggests a model in which many maternally contributed TFs show continued expression, and additional TF classes are subsequently enhanced or switched on to control tissue-specific expression programs. The role of the group of maternal TFs with a zf-AD whose expression diminishes later in development remains enigmatic, as only few of them seem to have an essential function or are evolutionarily conserved ([39] and references therein). The expression behavior of the other TF families makes sense in the light of the following observations: many developmental regulators that commit cells to specific lineages are members of the HLH TF family - for example, Twist is the master regulator of mesoderm development [40], Single minded of central nervous system midline development [41], and Trachealess induces the tracheal cell fate [42]. Homeodomain TFs, like the zinc finger TFs, contribute to axis determination (for example, Bicoid [43]) and segmentation (for example, Ultrabithorax [44]), but after specific lineages have been specified also facilitate differentiation processes (for example, Drifter [45]).

Other TF classes with little or no maternal contribution, whose number of expressed TFs increases during embryogenesis, are the aforementioned bZIP family, and the Forkhead, Ets, T-box, and GATA TF families. This expression behavior does not imply that individual TF family members are not important for early development. In fact the Sloppy paired TFs (with a Forkhead DBD) play a role in segmentation [46], Ets98B is important for pole cell migration [47] and Brachyenteron (with a T-box DBD) is involved in gastrulation and gut formation [48]. However, it is only in later stages that more members of these families are expressed during the formation of organ systems, as shown in Figure 1b. Examples of this are the Forkhead TFs Forkhead (hindgut and malpighian tubules [48], and central nervous system [49]), Binou (visceral mesoderm) [50], Jumeaux (neuroblasts) [51], and fd3F (sensory neurons) [52].

### TF expression peaks in gastrulation and the onset of organogenesis

**Figure 2:**
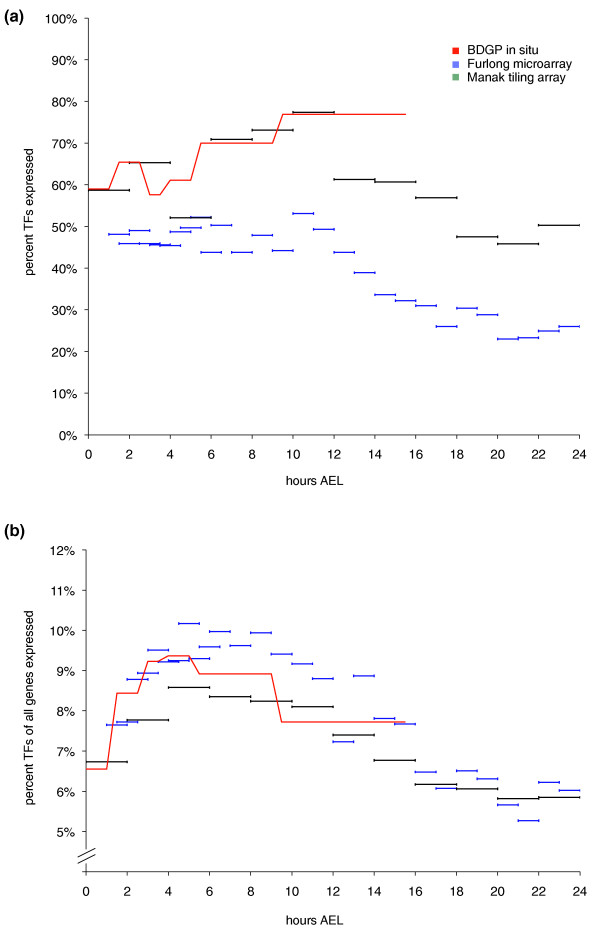
**Temporal utilization of site-specific transcription factors**. **(a) **Percentage of the TF repertoire used during embryonic development. Samples were taken at various degrees of granularity (red, BDGP *in situ *database; blue, embryonic microarray gene expression time-course; green, active transcription map). Although there are differences in the absolute number of transcribed TFs, there is good agreement in the general trends. About half of the TFs are maternally contributed. The peak of TF expression is around 10 to 12 h AEL, and the number of expressed TFs declines towards the end of embryogenesis. **(b) **Proportion of TFs in the group of expressed genes. The ratio of TFs versus non-TFs is highest between 2 and 6 h AEL. This coincides with the functional compartmentalization of the germ layers leading to the development of various organ systems.

The overall temporal expression of site-specific TFs is shown in Figure 2a. The BDGP dataset indicates that between 60% and 75% of the TFs are expressed over the time period 0 to 16 h AEL, with a peak at about 12 h AEL. The expression of TFs in the active transcription map of Manak *et al*. [27] closely follows the same trend. The active transcription map extends about 8 h beyond the BDGP, and during this period (12 to 24 h AEL) the percentage of expressed TFs drops steadily to between 45% and 50%. This general trend, including the maximum at 10 to 11 h AEL and a steep drop between 11 and 24 h AEL is confirmed in the Hooper *et al*. dataset. This means that the later stages, including the differentiation of organ systems, show a much more complex expression behavior than the *in situ *hybridization database may suggest.

Though in absolute terms the number of TFs expressed peaks at about 12 h AEL, the proportion of TFs relative to non-TFs expressed in embryogenesis peaks around 4 to 8 h AEL (Figure 2b). During this time, up to 10% of the genes expressed in the embryo are TFs, which is twice the percentage of TF genes amongst all genes in the fly genome [21]. Both the temporal gene expression array and the active transcription map confirm this trend, with small differences in the timing and intensity of the TF expression peak. In agreement with this, the expression classes 'II' and 'II:a' (showing transient expression) identified by Hooper *et al*. [28] fall into the 6 to 8 h AEL time frame, and these two groups have the largest proportions of TFs ('II', 10%; 'II:a', 13.5%) compared to their other classes. This peak in the proportion of TFs expressed coincides with gastrulation and the onset of organogenesis.

In all three datasets, the proportion of TFs expressed relative to non-TFs rapidly rises after the onset of zygotic expression, and continuously falls after stage 10 (BDGP database) or 6 to 8 h AEL (microarray experiments). This means that despite large numbers of TFs being expressed in embryonic tissues at later stages of development, the regulatory complexity in terms of the TF:non-TF expression ratio is greatest during the period of gastrulation and the onset of organogenesis.

### Transcription factor expression along the lineage of embryonic tissues

**Figure 3:**
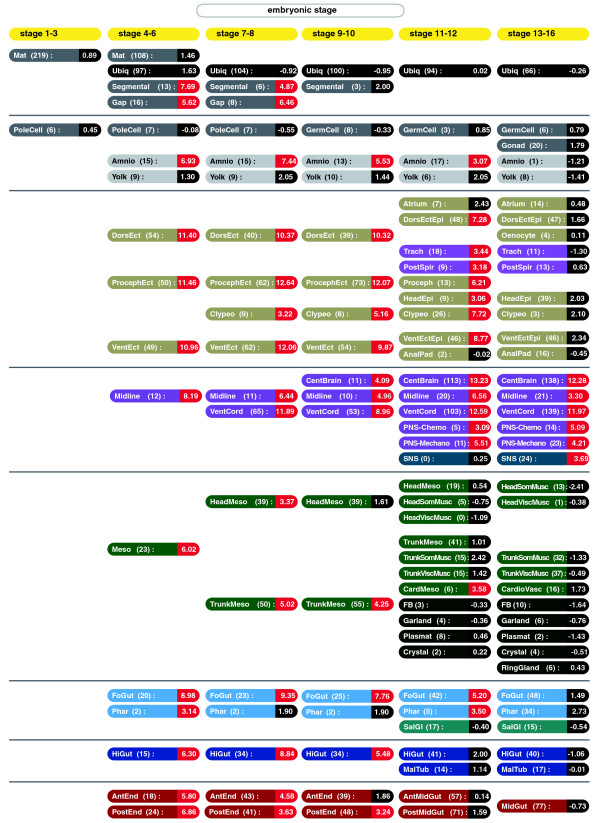
**Spatio-temporal over-representation of transcription factors in embryonic development**. This figure uses the slim representation of the anatomical ontology from Tomancak *et al*. [30] with the same color codes. The number of TFs expressed at the given spatio-temporal coordinate is provided in brackets, and the decimal number indicates the Z score for the over-representation of TFs in comparison to non-TFs. Statistically significant enrichment of TFs versus non-TFs (Z > 3) is highlighted in red.

In order to gain an overview of the spatial and morphogenetic component of TF expression during embryogenesis, we analyzed TF over-representation in various embryonic tissues (Figure 3). We calculated TF over-representation by comparing the proportion of TFs expressed relative to non-TFs with the genomic percentage of TFs (5% of all genes). The relative over-representation of TFs is quantified in terms of a Z score, with a value of about 3 or more being significant. The BDGP *in situ *database is the only spatio-temporal expression dataset, so further analysis is restricted to these data.

The maternally contributed transcripts exhibit the lowest fraction of TFs relative to all transcripts (Figures 2b and 3). Stages 4 to 8 see a sharp increase in this percentage, with over-representation of TFs in all tissues except the yolk and the pole cells. The yolk and the developing germ cells continue to express a low proportion of TFs in later embryonic development. This is in agreement with the yolk as a storage tissue, and the transcriptional quiescence of pole cells [53]. The other tissue that has a diminishing proportion of TFs is the amnio serosa; the degree of TF over-representation falls throughout embryogenesis to stages 13 to 16, when it only expresses one TF. This makes sense as it is an apoptotic tissue, which probably requires less regulation towards its end.

In stages 4 to 8, there is significant regulatory complexity in all the different germ layers and their derivatives. The ectoderm and the mesectoderm show the highest degree of TF involvement, reflecting the complex developmental competence of these germ layers. The mesoderm at stages 4 to 6 and endoderm at stages 4 to 6 exhibit a reduction of TF over-representation at later stages of development. In fact, at stages 9 to 10, only the trunk mesoderm and the posterior endoderm show significant TF enrichment. After stage 10, only the cardiac mesoderm has significant TF involvement.

This expression behavior is contrasted by a stable TF enrichment in most ectodermal (for example, procephalic ectoderm, dorsal ectoderm, and foregut anlage and primordium) and mesectodermal tissues (mesectoderm primordium, and ventral nerve cord anlage and primordium) into stages 11 to 12. This is in keeping with the findings of Reece-Hoyes et al. [35], who observed similar patterns in the worm *C. elegans*.

In the process of further differentiation, only the nervous system (for example, central brain, and peripheral nervous system) maintains high levels of TF enrichment, whereas TF over-representation in the dorsal and ventral epidermis and the tracheal system diminishes from stage 13 onwards. These results are in keeping with the pattern observable in Figure 2b.

The transcriptional programs necessary to initiate gastrulation and organogenesis cause the peak of TF over-expression. In the later embryonic stages of organ system differentiation, the high degree of TF involvement is limited to the nervous system. This is in agreement with the finding of Tomancak *et al*. [30] that many TFs are involved in nervous system development, and TFs are over-represented in these tissues. The exceptional position of the nervous system compared to all other tissues is especially interesting in the light of absolute TF numbers. Indeed, the number of TFs is highest in the embryonic brain (138) and the ventral nerve cord (139). Even the mechanoreceptors of the peripheral nervous system have significant over-representation of TFs (23 TFs). Perhaps the number of different cell types or spatial complexity influence the degree of TF over-representation, as the nervous system and the brain in particular have the highest diversity and structural complexity of all organs (see [54] for review).

### TF genes are expressed more tissue-specifically than other genes

**Figure 4:**
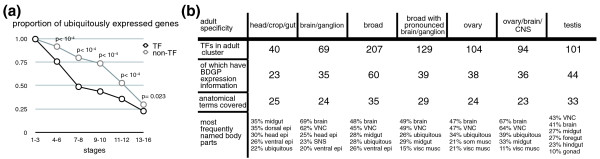
**Tissue specificity of site-specific transcription factors**. **(a) **Proportion of ubiquitously expressed genes along the developmental time axis, separated into TFs and non-TFs. The expression patterns of TFs and non-TFs are significantly different between stages 4 and 12 (*P *< 10^-4^), with less ubiquitous and more restricted expression patterns for the TFs. **(b) **Adult specificity. Gene expression clusters with adult tissue-specificity were queried for their specificity in late embryonic development (BDGP dataset stages 13 to 16). Shown is the expression breadth as the number of anatomical structures used in the annotation, as well as the percentage of genes with frequently encountered annotations. For example, for the 40 TFs primarily expressed in adult head, crop and gut, there is embryonic expression information for 23 of them. These genes are most frequently annotated for expression in the midgut (35%) and dorsal epidermis (35%). Interestingly, very frequently, adult tissue-specific TFs are involved in embryonic nervous system development. CNS: central nervous system; SNS: stomatogastric nervous system; VNC: ventral nerve cord.

Having surveyed TF expression in the developing embryonic tissues, we were interested to gain insight into the difference between TFs and non-TFs. Figure 4a shows that both groups have a substantial fraction of ubiquitously expressed factors and increasing specificity towards the later stages of development. However, the proportion of TFs with specific expression is significantly larger than that of non-TFs between stages 4 and 12 (Figure 2a). Although 146 TFs in the dataset are ubiquitously expressed at some stage during development, only 19% (28) of them exhibit exclusively ubiquitous expression. In other words, most of them have restricted expression at some stage of development.

We were interested to see if these trends were alike for all of the major TF classes or if the apparent difference between the TFs and the non-TFs was primarily driven by a particular TF class. All of the large TF classes with at least about 20 members follow the previously described trends. Interestingly, the classes that we have discussed here with special importance in organogenesis (for example, Homeobox, HLH, Forkhead, bZIP1 and bZIP2) show the strongest tendency for specific expression.

'Specific' expression for most TF classes implies restriction to about one-quarter of terms used in the slim representation of embryonic anatomy. To further explore this finding, we were interested to see how narrow TF expression can be. When we excluded all TFs that are ubiquitously expressed at any one embryonic stage, we found that only about 10% of the remaining TF genes are expressed in less than three anatomical structures. Factors with expression in clearly different tissues with respect to developmental origin or function were manually excluded. The largest uniform group retained in this analysis is a set of 12 TFs expressed exclusively in the central brain and the ventral nerve cord (for example, Sox102F and a few previously uncharacterized factors), and the second largest comprises 5 TFs in the trunk musculature and related mesodermal structures (for example, Lame duck). Other tissues exhibit one or two exclusive TFs (for example: midgut, Estrogen-receptor related and Adult enhancer factor-1; head epidermis, PvuII-PstI homology 13; yolk, Cryptocephal). However, the latter gene also highlights the general difficulty of such analysis. While the BDGP annotators note a yolk-specific staining, a detailed study of the gene finds *cryptocephal *ubiquitously expressed [55].

### Tissue-specific expression in the embryo versus the adult

As discussed above, most TFs expressed in the embryo are not specific to one but several tissues. Next, we ask whether this specificity in the embryo relates to expression of the TFs in the adult tissues of the FlyAtlas. In the adult, most TFs are broadly expressed, independent of what definition of expression is used to analyze the FlyAtlas data (Additional files 5 and 6). About two-thirds of the 687 TFs present in the adult show ubiquitous or very broad expression based on the FlyAtlas 'present' call (Additional file 6).

We then used the 'up' call to identify more tissue-specific adult TF expression as this should discern genes with significant expression in the respective body parts (Additional file 6). We identified seven clusters of TFs and classified them according to the adult tissues where they are expressed, as shown in Figure 4b. These seven clusters correspond to expression in ovary, testis, brain/ganglion or combinations of tissues (Additional file 6). The adult TF clusters with broad or ovarian expression do exhibit a broader embryonic expression than the groups with some adult specificity. However, although the adult tissue-specific clusters are also tissue-specific in the embryo, this does not necessarily pertain to the same or comparable tissue. This shows that adult TF expression is largely independent of embryonic expression. A good example is Trachealess, which is an inducer of the tracheal cell fate that is also expressed in the embryonic salivary gland, but also plays a role in the formation of the adult leg [56]. Tissue-specificity in terms of expression breadth is a property that seems inherent for some TFs; however, the tissues where they are expressed can be rather diverse and variable between embryo and adult.

### Combinatorial use of transcription factor modules

Both anatomical complexity and absolute number of expressed TFs increase along the developmental time axis. At the same time, the tissue-specific expression of the TFs increases, ultimately leaving many differentiating organ systems except the nervous system with a significantly lower TF:non-TF ratio than, for example, their anlagen and primordial tissues. In addition, we show above that absolute tissue-specificity is virtually nonexistent. All this supports the concept that developmental and physiological gene expression programs are not regulated by individual factors, but by the unique combination of TFs present in a given biological context.

**Figure 5:**
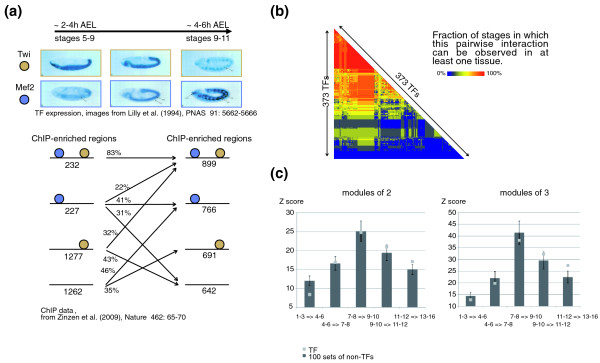
**Modularity of transcription factors**. **(a) **Expression of Twi and Mef2 and their co-occurrence at genomic sites at different developmental stages. Compared are stages 5 to 9 (approximately 2-4 h AEL) and stages 9 to 11 (approximately 4-6 h AEL). In the earlier time frame, strong mesodermal Twi expression but only weak Mef2 expression can be observed. Half of the genomic sites that see occupancy by either TF at some stage during development show only Twi occupancy (43%) or Twi/Mef2 (8%) double-occupancy. By 4 to 6 h AEL, the majority of the mesoderm develops into muscle, which is characterized by strong Mef2 expression. Genomic occupancy follows this trend, showing that most sites with previous double-occupancy maintain that state while one-third of the previous Twi-only sites gain a Mef2 partner site. Overall, more than half of the Twi sites then show double-occupancy. The images showing TF expression are reproduced from [64]. ChIP data are from [22]. **(b) **A map of potential pairwise interactions between transcription factors. For all of the 373 TFs for which BDGP expression data are available, the frequency of co-occurrence in at least one tissue is color-coded (as fraction of sampled time frames). Most TFs are co-expressed in at least one spatio-temporal coordinate. **(c) **Transcription factor expression exhibits modular behavior. More modules of two (left panel) or three (right panel) transcription factors show precisely the same expression from one to the next developmental stage than is to be expected at random. Interestingly, this is an intrinsic feature of all genes and not a specific property of the transcription factors. Error bars indicate the standard deviation observed in the random experiments.

In order to quantify the combinatorial usage of TFs in embryonic tissues, we trace the co-expression of pairs and triplets of TFs across developmental stages and tissues. Examples of this principle are Twist and Mef2, two co-expressed TFs that collaborate in muscle development. Temporal genome-wide binding data are available for the master regulator of mesoderm development, Twist [18], and the inducer of myogenesis, Mef2 [57]. The comparison of Twi and Mef2 occupancy shows that most Twi sites present at 2 to 4 h AEL are only occupied by Twi (85%) and do not see Mef2, the earlier expressed of the two factors. When mesodermal differentiation continues and Mef2 expression levels increase, previously double-occupied sites maintain that state whilst about one-third of the Twi-only sites gain double-occupancy at 4 to 6 h AEL (Figure 5a). This shows that although TFs do act as cooperative modules, binding at *cis*-regulatory sites is extremely dynamic, and in some cases directly reflects TF gene expression.

In line with this principle, we calculated how many of the possible pairs of TFs are expressed during embryonic development. From the BDGP data, the possible number of co-expressed TF pairs would be about 69,500 (373^2^/2). In Figure 5b, we show that the vast majority of these possible pairs are in fact co-expressed in at least one tissue and developmental stage. This means that they can potentially collaborate at genomic binding sites, an assumption that may be especially true if these TFs are persistently co-expressed in exactly the same tissues.

Next we ask how many of the co-expressed pairs and triplets of TFs are maintained as co-expressed modules across consecutive stages of embryonic development. In Figure 5c, we show that there is highly significant conservation of co-expressed pairs and triplets of TFs from one stage to another compared to a set of TF genes whose expression information has been randomly shuffled. However, similar levels of maintenance of co-expression are found in non-TFs. The only embryonic stages that show significantly higher conservation of co-expressed TF pairs and triplets compared to non-TFs is the transition from stages 11-12 to 13-16.

This analysis shows that overall co-expression of TFs is extremely plastic, with almost the entire space of possible TF pairs co-expressed at some point in embryonic development. Co-expression TF modules, however, represent potential functional entities that are much more abundant than to be expected at random. There is no evidence for these consistently co-expressed TFs to interact at *cis*-regulatory modules, even though it is known that TFs cooperate, as in the example of Twist and Mef2 above.

## Conclusions

Functional genomics approaches provide unbiased insight into the spatial and temporal utilization of *Drosophila *genes on a genome-wide level. We integrated these data with information about the repertoire of site-specific TFs in the fly, and studied their expression dynamics in order to understand their role in development and physiology on a systems level. A potential shortcoming of this approach is that we are considering RNA levels as a proxy for TF activity. Because there are so few data available at the protein level, we are obliged to use the mRNA data. While mRNA levels will not always reflect protein levels, at least both the microarray and *in situ *datasets are relatively robust to transcriptional noise. This is achieved by both technical and empirical optimization of these methods.

A further limitation of both types of expression dataset is the granularity of both the sampling and the annotation. This implies that many of the co-expression clusters at the level of tissues may not actually be co-expressed at the level of individual cells. Therefore, our conclusions about re-shuffling of TFs between clusters may be even further enhanced once more fine-grained expression data become available.

Our data suggest that at least 50% to 60% of the TFs are maternally contributed (Figures 1 and 2a), which is in keeping with previous studies on the ascidian *Ciona intestinalis*, where a similarly high proportion was reported [58]. The early *C. elegans *embryo expresses only about 30% of its TFs [35], and it can be speculated the pre-defined developmental cell lineages do not require the same large number of maternal TFs. These studies are the only other works that address TF utilization in metazoan organisms on a genome-wide level, and therefore serve as an interesting comparison.

The number of early/maternal TFs is much larger than could be assumed based on a dozen or so anecdotal early patterning genes. This is a very important finding in the light of studies of early gene regulation. Early patterning in *Drosophila *is often viewed as a closed circuit of a handful of TFs regulating each other [1], and most of the recent network and perturbation analyses are based on this assumption. How does the plethora of maternally contributed TFs tie into the known and well-understood network? Recent studies suggest that the genome is plastered with binding sites occupied by the well-known early factors in blastoderm embryos [19]. Is this how they differ from the less prominent bulk of maternal TFs? Or is the early genome covered by hundreds of TFs, waiting in place to fulfill their function at a later stage?

The fraction of TFs utilized during embryonic development is about 95%, both in our study and in the analysis of *Ciona *TFs [58]. Given that these results are obtained by a variety of different assays (*in situ *hybridization, microarray) and in two different model organisms, this is unlikely to be an experimental artifact. The general time-course of TF expression suggests that the largest degree of combinatorial complexity occurs between 4 and 12 h AEL (Figure 2b), coinciding with the formation of germ layers and the onset of organogenesis. This is when the ratio of TFs to non-TFs is highest. Interestingly, the actual peak of TF expression is only achieved later in development, when both the number of body parts and TF specificity increase. Expressed TF numbers fall from 12 h AEL (that is, stage mid-15) onwards (Figure 2a), suggesting that the final differentiation of organ systems is a local event requiring less TFs per tissue. In the earlier phases of development large numbers of TFs seem to be required, either for inter-regulation or combinatorial regulation of target genes. The coding potential of fewer TFs appears to be sufficient once the embryo has been compartmentalized.

In this work we show that the Homeobox TFs make a large contribution to these local expression neighborhoods in late embryogenesis (Figure 1b; Additional file 4). Recent studies on the coding limits of TFs show that Homeobox TFs have by far the largest number of possible binding sites [59], which also supports the fact that a smaller number of TFs may achieve the same coding potential. Similar findings to our results regarding temporal utilization of TF families can also be found in *Ciona*, with Homeobox and HLH families generally showing a later onset of expression. This is in agreement with the developmental role these classes have been associated with: zinc finger TFs are a versatile class, while many other TF families (for example, HLH, Homeobox, T-box, Forkhead) are over-represented in organogenesis and differentiation (Additional file 7). This role is also reflected in their developmental expression, with zinc finger TFs showing ubiquitous expression more frequently than other classes (data not shown). This contrast is particularly strong in the light of TF overlap between tissues, where we show that zinc finger TFs are more likely to be shared between tissues than Homeobox TFs (Additional file 8).

We were surprised to see a general lack of true tissue-specificity, although TFs often have narrow expression domains. This is especially highlighted by the comparison of adult tissue-specificity and embryonic expression (Additional file 6; Figure 4b), where we found that those TFs that show some adult tissue-specificity are expressed in unrelated tissues during embryonic development. We did observe a biologically sensible correlation, however, between TFs expressed in the ovary and those present as maternal contribution. As many maternal TFs seem to play a role in embryonic nervous system development, there is also a group of TFs that show specific adult expression in the ovary and brain/central nervous system. Interestingly, many factors of the enigmatic group of zinc finger TFs with a zf-AD have this pattern, a group that shows strong lineage-specific expansion in holometabolous insects [39].

The combinatorial usage of site-specific TFs is an established concept. However, most of our current knowledge is based on a few examples like the 'stripe 2' enhancer [10]; we are only slowly accumulating sufficient data to address the combinatorial characteristics of regulatory modules on a broader scale [19]. Many previous studies concentrated on the inference of combinatorial TF usage from the co-occurrence of binding motifs [60,61], but the expression of the TFs themselves was mostly neglected. Context-specific co-expression is one possibility for how combinatorial TF usage can be studied. While many TFs can be present in a particular tissue, the most consistent co-expression clusters are formed by TFs whose pattern precisely correlates in a variety of different tissues at a given stage. These clusters are rather dynamic and rarely stable between stages, and TFs are constantly exchanged between co-expression clusters along the developmental time axis (Figure 5c). However, they are more frequent than can be explained by chance and probably a good indicator of functional relevance. The characteristics of this reshuffling process have not been addressed on a systems level before, and it should be noted that their expression modularity is not much stronger than for non-TFs. This implies that a 'transcriptional logic' is probably not primarily conferred by the expression of TFs, as studies on early patterning might suggest.

Taken together, our study provides a first analysis of the system-wide behavior of *Drosophila *TFs. Our findings support the existence and predict a developmental role for almost the entire TF repertoire. The tissue-specific role of the HLH and Homeobox TF families, which is known anecdotally for a handful of genes, is supported by high-throughput gene expression data on a genome-wide level. The defined expression of TFs contributes to a constant reshuffling of TFs between clusters of co-expression, providing the outer bound for TF combinatorics. Expression modularity is not an inherent feature of TFs to a greater extent than non-TFs, which has implications for their regulatory logic.

## Materials and methods

### Gene expression datasets

Spatio-temporal gene expression was derived from the BDGP gene expression database [29,30], pre-release version as of May 2006, using the *Drosophila *anatomical ontology or the slim representation of Tomancak *et al*. [30] to map individual body parts to developmentally related lineages. The BDGP contains information on 373 TFs, 51% of the TF repertoire in the FlyTF database [21].

Temporal gene expression information was derived from an embryonic gene expression time-course genome-wide microarray experiment [28], a microarray survey of early embryonic expression [32] and a time-course genome map of active transcription during embryonic development [27]. The transcript abundance per time point in the time-course gene expression experiment is relative to a pool of mRNA of all developmental stages, including larval, pupal and adult stages. Genes with no developmental change or those that are expressed at lower levels in the embryo than in other stages are therefore indistinguishable from genes that are not present in the embryo. Expression classes as observed by Hooper *et al*. [28] using a global convolution method were accessed through FlyMine [62] and overlap was determined using the FlyMine list manager. We chose an additional strategy to classify transcript abundance, and inferred three arbitrary classes ('low expression', value < -0.5; 'not classifiable/average', -0.5 < value < 0.5; and 'high expression', value > 0.5). These thresholds are based on the observation that the median expression value per time point is 0.72 for BDGP positive and 0.14 for BDGP negative genes, with a considerable spread of the data not allowing for more defined thresholds.

In the Affymetrix GeneChip experiment of early development by Pilot *et al*. [32] we counted a gene as expressed when it received at least two present calls in the three replicates. The 'present' call is based on the statistical analysis of all probes per gene and provides an indication of whether the observed signal stems from a transcript (if it is present or absent) or is part of a general background. We interpreted overlap of at least 10% of a gene's coding region with any transfrag from the active transcription map as a sign of its expression. This is less stringent than the criteria used by Manak *et al*. [27], who required at least 70% of a gene's coding sequence to be transcribed. However, lower transfrag coverage may simply be due to technical issues when the transcript is present at low abundance. Overall, our criteria produced similar results between the three temporal gene expression datasets.

Adult gene expression information was derived from FlyAtlas [31]. We rated a gene as expressed when it had at least three present calls in the four replicates per adult tissue, or if it was significantly up-regulated (received an 'up' call) in a tissue when compared to whole flies.

Additional file 9 provides a convenient lookup for the conversion between developmental time (hours AEL) and developmental stage.

### Transcription factor over-representation in body parts

We used a slim representation of the anatomical ontology, using the tissue categories defined by Tomancak *et al*. [30], to allow comparability between the studies. We determined the non-redundant set of TFs (n) and non-TFs (m) expressed in body parts represented in each tissue category. Random sampling of n + m genes was used to calculate the number of TFs to be expected by chance, and a Z score was derived. We considered TFs to be over-represented in a tissue if Z ≥ 3. This approach is comparable with the anatogram summaries of Tomancak *et al*., where Z is the amplitude of the signal.

### Significance of transcription factor overlap

The significance of TF repertoire overlap (Additional file 8) between two tissues with n and m TFs was determined by random simulation, comparing the actual overlap with overlaps obtained by randomly picking n and m genes from all TFs of the appropriate family with expression information.

### Muscle transcription factor reshuffling

ChIP datasets [22] were obtained from the Furlong laboratory website. Filtered TileMap data were used to indicate reliable binding of Twi and Mef2.

There are no generally accepted criteria as to what defines the boundaries of a *cis*-regulatory module or TF binding site cluster. We therefore interpreted partial or complete overlap of ChIP peaks as an indication of common action of TFs within a genomic region.

### Modularity

We interpreted precise co-expression as a minimal requirement for modularity. If two or three TFs are consistently used in exactly the same developmental context (that is, precisely co-expressed at subsequent developmental stages), then they potentially form a module. We counted the potential pair-wise or triplet interactions between precisely co-expressed TFs in one stage and compared them to the potential interactions of the previous stage. The number of 'conserved' interactions between the two stages was counted. In a random experiment we used the same pairing matrix of the older stage, but each time reshuffled the order of the TFs for each of 1,000 iterations. In each of the iterations, we determined the number of 'conserved' interactions, which we used to calculate a Z score in comparison with the real data. These data were compared to the Z scores obtained by randomly sampling 373 genes and repeating this approach 100 times.

### Gene Ontology analysis

Lists of candidate genes were uploaded to FlyMine and GO enrichment was analyzed using their GO widget with 'Benjamini and Hochberg' correction. For GO over-representation within the TFs, GO annotation was downloaded from FlyMine and over-representation for TF classes in comparison to a background of all TFs was calculated in GeneMerge v1.2 [63].

## Abbreviations

AEL: after egg lay; BDGP: Berkeley *Drosophila *Genome Project; ChIP: chromatin immunoprecipitation; DBD: DNA-binding domain; GO: Gene Ontology; GRN: gene regulatory network; HLH: helix-loop-helix; TF: transcription factor; zf: zinc finger; zf-AD: zinc finger-associated domain.

## Authors' contributions

BA and SAT designed the study, analyzed the data and wrote the manuscript.

## Supplementary Material

Additional file 1 **Size of transcription factor families in *D. melanogaster***. The classification is based on the DBD present in the TF. Shown are TF families with at least five members. It is noteworthy that these 14 families (of about 50 TF families encoded in the fly genome) account for approximately 70% of all site-specific TFs. The largest TF class uses the C2H2 zinc finger for DNA binding. It is also the class with the highest degree of uncertainty in terms of its function, as this zinc finger type can also be involved in RNA binding or protein interactions. Functional assignment on the bases of other DNA-binding domains is of much higher confidence. Click here for file

Additional file 2 **Mini-website: raw data and intermediate results**. A self-contained website to browse and retrieve all primary data used in this study, as well as intermediate results such as clustering results. Click here for file

Additional file 3 **More details on expression classes a and d**. A PDF file containing more details on expression classes a and d. Click here for file

Additional file 4 **Utilization of TF families during embryonic development**. **(a) **Distribution of DNA-binding domains amongst the four expression groups indicated in Figure 1a. There are clear differences in the utilization of DNA-binding domains along the developmental time axis. While C2H2 zinc finger TFs show both early and later expression, other classes such as Homeobox or HLH TFs are primarily expressed in the later stages. Therefore, the relative abundance of zinc fingers is much higher in the group of early TFs, whilst the other classes take over later on. **(b) **Relative proportion of TF family usage according to microarray-based approaches (top, active transcription map; bottom, expression time-course) with a finer degree of resolution. The trends seen in the BDGP *in situ *database are confirmed by the unbiased microarray approaches. Click here for file

Additional file 5 **TF versus non-TFs in adult tissues**. The tables list the number of expressed genes and how many of them are TFs. For each expression criteria and adult tissue, a Z score is given. In summary, there is no significant over-representation in adult tissues as can be observed during embryonic development. Click here for file

Additional file 6 **Clustering of TFs according to their adult tissue specificity**. Different criteria for specificity alter the outcome. **(a) **Criteria: 'present' call in at least three of the four replicates. Almost half of the TFs are ubiquitously expressed. **(b) **Criteria: 'up' and 'down' call in respect to whole fly material. This definition allows identification of distinct clusters of specificity (as used in the analysis of Figure 3b). The clusters are named according to the tissues where there is the largest number of 'up' calls. Click here for file

Additional file 7 **Enrichment of GO categories for specific TF classes against a general TF background**. The tables show GeneMerge output for the main TF classes. The three GO main hierarchies were summarized into one table per TF class. GO annotations with raw_es < 10^-3 ^were manually inspected for developmental roles. While all TF classes show the most significant enrichment for their role in transcriptional regulation, the zinc finger TFs do not show specific enrichment for any developmental process, as it is the case for, for example, the Homeobox TFs. Click here for file

Additional file 8 **Overlap of TF repertoires**. The degree of overlap is presented as a Z score, which can be interpreted as a correction for repertoire size. Clustering of these scores groups body parts with particular similarity together, and separates, for example, the ectoderm from the mesoderm. Interestingly, negative Z scores that can be interpreted as an avoidance of overlap exist mostly for the ubiquitous maternal and ubiquitous zygotic TFs. The smaller panels on the right side show the same analysis for the largest TF families (black indicates the lack of body parts expressing TFs of the family). Importantly, these results argue that zinc finger TFs are shared more frequently between tissues than, for example, Homeodomain TFs. Each panel reads as the overlap from the perspective of the tissues along the top to the tissues along the side (these can be different because of the TF repertoire sizes of the respective tissues). Click here for file

Additional file 9 **A comparison of developmental time and developmental stage in *Drosophila***. A PDF file showing comparison of developmental time and developmental stage in *Drosophila*. Click here for file
