# Supplementary figures and images for: The developmental expression dynamics of Drosophila melanogaster transcription factors
Source: Genome Biol. 2010 Apr 12;11(4):R40. doi: 10.1186/gb-2010-11-4-r40 (PMC2884543; doi:10.1186/gb-2010-11-4-r40)

## Adryan\_Supplementary Figure 1

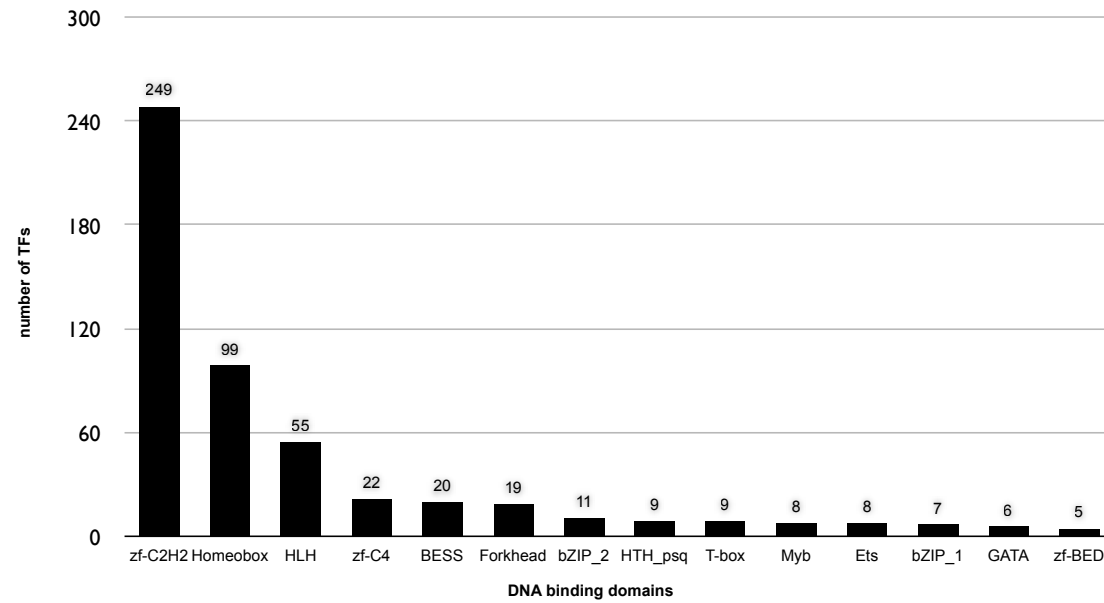

Supplement: Additional file 1 — Size of transcription factor families in D. melanogaster. The classification is based on the DBD present in the TF. Shown are TF families with at least five members. It is noteworthy that these 14 families (of about 50 TF families encoded in the fly genome) account for approximately 70% of all site-specific TFs. The largest TF class uses the C2H2 zinc finger for DNA binding. It is also the class with the highest degree of uncertainty in terms of its function, as this zinc finger type can also be involved in RNA binding or protein interactions. Functional assignment on the bases of other DNA-binding domains is of much higher confidence. [file gb-2010-11-4-r40-S1.PDF]

# Adryan\_Supplementary Figure 2

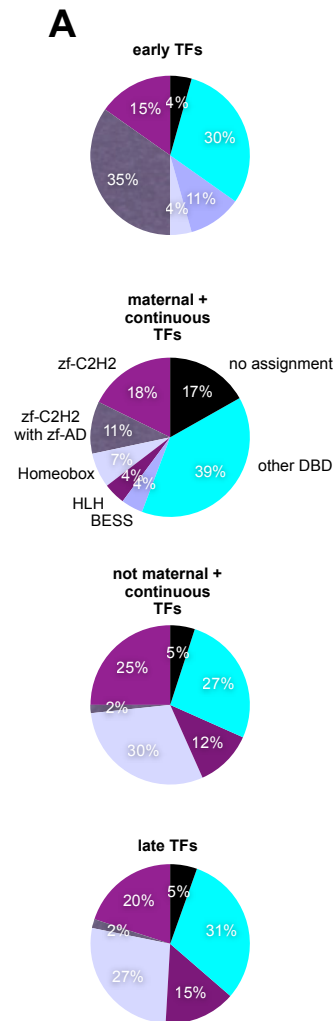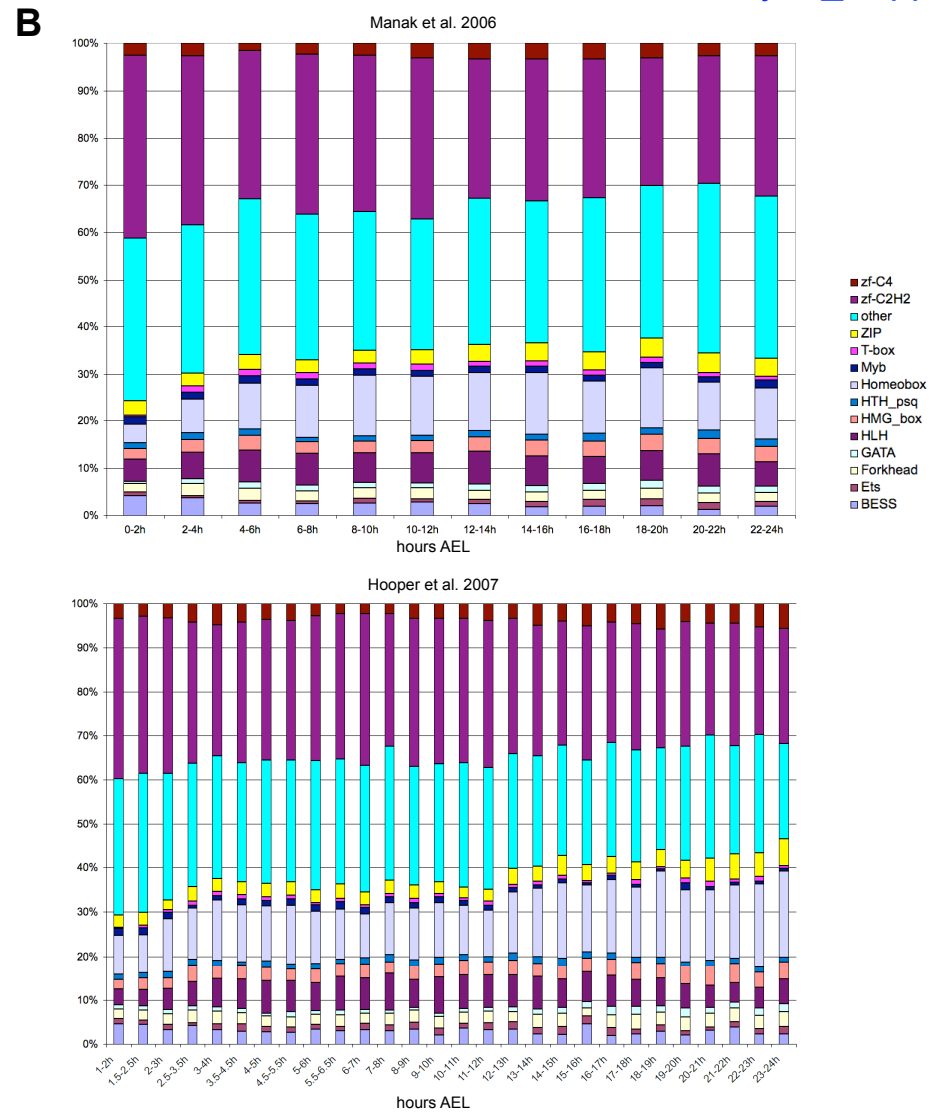

Supplement: Additional file 4 — Utilization of TF families during embryonic development. (a) Distribution of DNA-binding domains amongst the four expression groups indicated in Figure 1a. There are clear differences in the utilization of DNA-binding domains along the developmental time axis. While C2H2 zinc finger TFs show both early and later expression, other classes such as Homeobox or HLH TFs are primarily expressed in the later stages. Therefore, the relative abundance of zinc fingers is much higher in the group of early TFs, whilst the other classes take over later on. (b) Relative proportion of TF family usage according to microarray-based approaches (top, active transcription map; bottom, expression time-course) with a finer degree of resolution. The trends seen in the BDGP in situ database are confirmed by the unbiased microarray approaches. [file gb-2010-11-4-r40-S4.PDF]

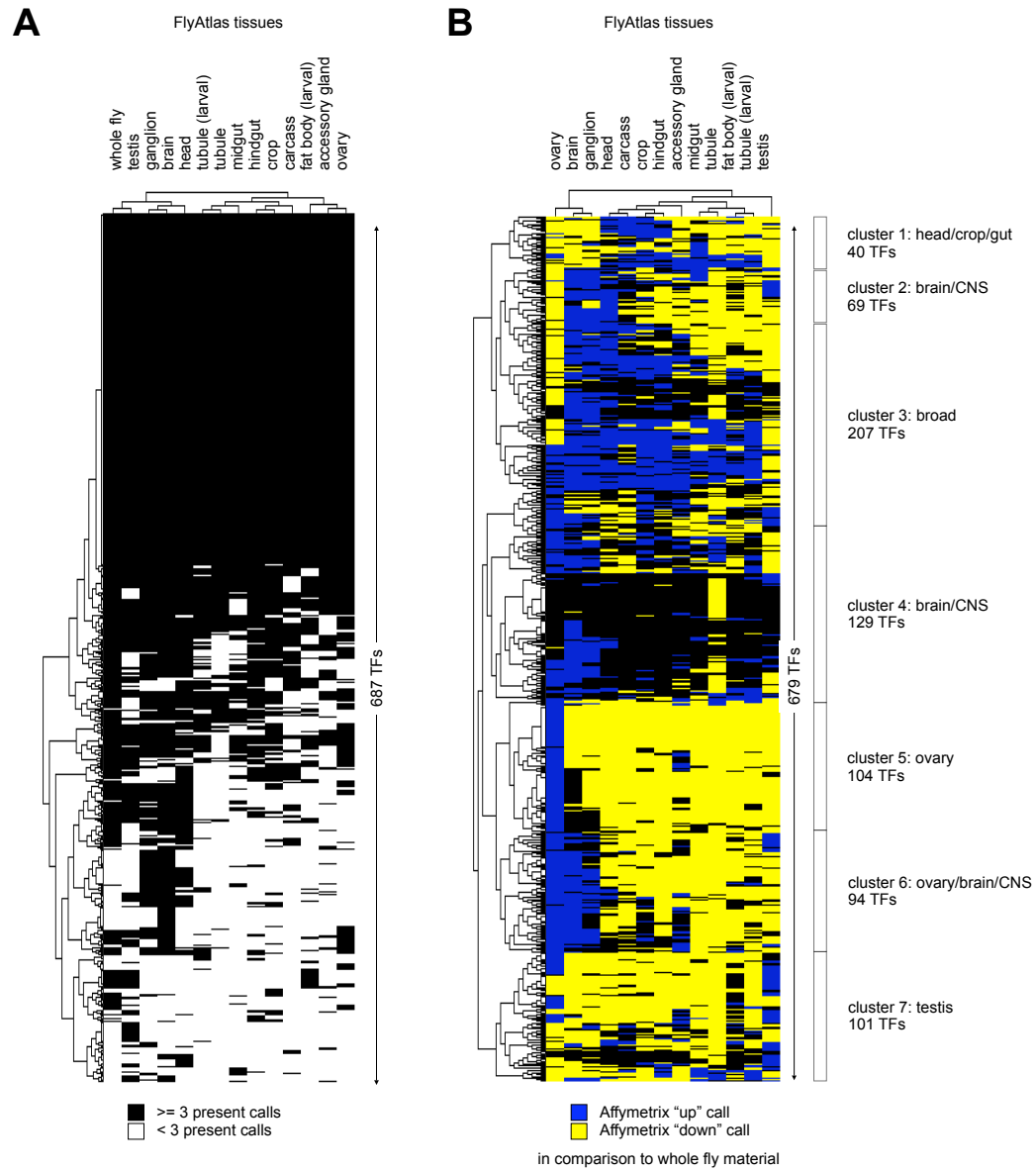

Supplement: Additional file 6 — Clustering of TFs according to their adult tissue specificity. Different criteria for specificity alter the outcome. (a) Criteria: 'present' call in at least three of the four replicates. Almost half of the TFs are ubiquitously expressed. (b) Criteria: 'up' and 'down' call in respect to whole fly material. This definition allows identification of distinct clusters of specificity (as used in the analysis of Figure 3b). The clusters are named according to the tissues where there is the largest number of 'up' calls. [file gb-2010-11-4-r40-S6.PDF]

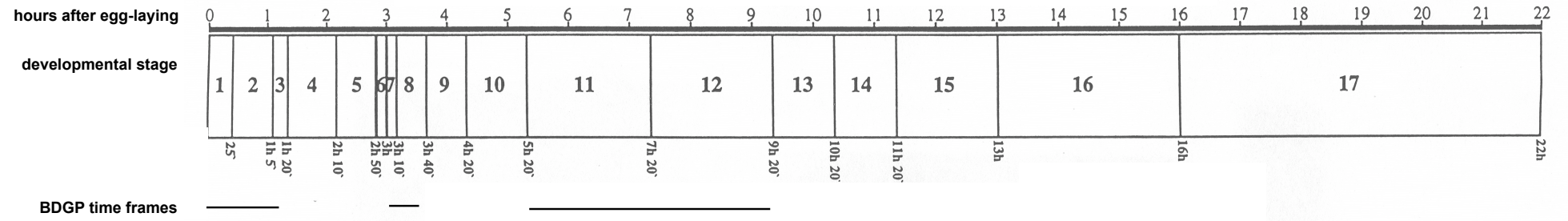

Supplement: Additional file 7 — Enrichment of GO categories for specific TF classes against a general TF background. The tables show GeneMerge output for the main TF classes. The three GO main hierarchies were summarized into one table per TF class. GO annotations with raw_es < 10-3 were manually inspected for developmental roles. While all TF classes show the most significant enrichment for their role in transcriptional regulation, the zinc finger TFs do not show specific enrichment for any developmental process, as it is the case for, for example, the Homeobox TFs. [file gb-2010-11-4-r40-S7.PDF]

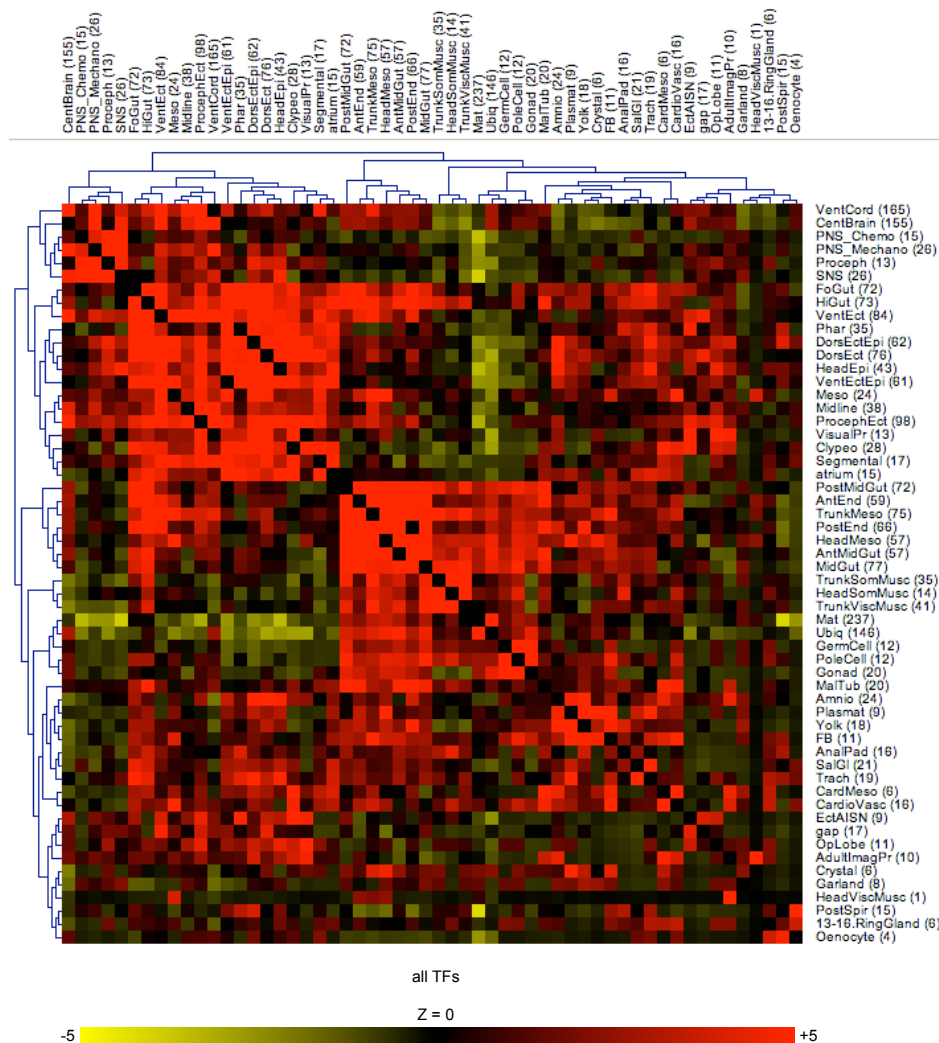

Adryan\_Supplementary Figure 3

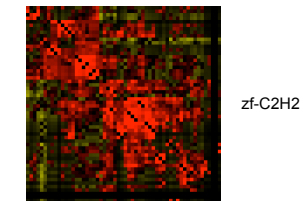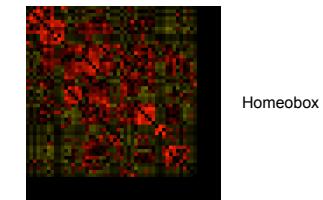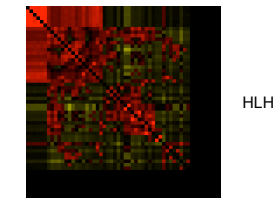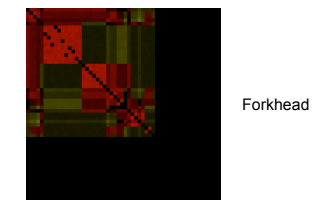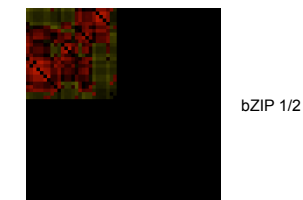

Supplement: Additional file 8 — Overlap of TF repertoires. The degree of overlap is presented as a Z score, which can be interpreted as a correction for repertoire size. Clustering of these scores groups body parts with particular similarity together, and separates, for example, the ectoderm from the mesoderm. Interestingly, negative Z scores that can be interpreted as an avoidance of overlap exist mostly for the ubiquitous maternal and ubiquitous zygotic TFs. The smaller panels on the right side show the same analysis for the largest TF families (black indicates the lack of body parts expressing TFs of the family). Importantly, these results argue that zinc finger TFs are shared more frequently between tissues than, for example, Homeodomain TFs. Each panel reads as the overlap from the perspective of the tissues along the top to the tissues along the side (these can be different because of the TF repertoire sizes of the respective tissues). [file gb-2010-11-4-r40-S8.PDF]
