# Supplementary material for: The developmental expression dynamics of Drosophila melanogaster transcription factors
Source: Genome Biol. 2010 Apr 12;11(4):R40. doi: 10.1186/gb-2010-11-4-r40 (PMC2884543; doi:10.1186/gb-2010-11-4-r40)
Supplement: Additional file 2 — Mini-website: raw data and intermediate results. A self-contained website to browse and retrieve all primary data used in this study, as well as intermediate results such as clustering results. [file gb-2010-11-4-r40-S2.ZIP › index.html]

Mini-website


# The developmental expression dynamics of Drosophila melanogaster transcription factors: raw data and intermediate results

Raw data are provided as comma separated values (.csv) with string
fields encapsulated in quotes, or as tab-delimited files (.txt) without
quotes. These files can easily be parsed with any scripting language,
or opened with recent versions of MS Excel.  
  

## Transcription factors

The list of 753 TFs under investigation is here: TF\_list.csv  
  
Not all of the 753 TFs could be mapped to transcripts. The list of 731
TFs for which there is also a CG identifier is here: TF\_w\_transcripts.csv  


---

  
The structural domain assignments (PFAM
and SUPERFAMILY)
used
in our analysis can be found here: TF\_domain\_assignments.csv.
Domain
assignments were derived using our internal pipeline detailed at
the DBD transcription
factor database, and qualifying DNA-binding domains are listed in
their download
section.  


---

## Expression data

### BDGP spatio-temporal gene expression

Spatio-temporal gene expression is based on Release 2 of the BDGP Gene Expression
Database, and we would like to acknowledge Pavel Tomancak for
making their data available prior to publication. The anatomical
ontology used in their work is available in terms.csv
and term-to-term.csv, and based on
this we have constructed a simplified representation of the tissues in slim\_term.csv.
Their
most recent data is available for download
here. The spatio-temporal expression matrix of transcription factors
(for the 373 TFs we found present in their dataset) is here: BDGP\_TF\_expr.txt.
Gene
groups of tissue-specific expression as defined in Table 1 can be
retrieved here: TFs from Table 1.  
  
The extracted temporal information is here: BDGP\_TF\_when.txt,
and
loading it along with BDGP\_TF\_when.xml into Genesis
provides our clustering result shown in Figure 1.  

### Microarray temporal gene expression

TF gene expression values (ratio of embryonic mRNA compared to a pool
of mRNA across all developmental stages) from the developmental time
course published by the Furlong laboratory (Hooper et al. 2007) is
here: Hooper\_time\_course.txt.  


---

  
Our dichotomised expression call based on transfrag data by Manak et
al. (2006) is here: Manak\_time\_course.txt. The
amount of exonic transcription per TF and time frame is available here:
Manak\_transfrag\_raw.txt.  


---

  
Affymetrix calls (present/absent) for TFs expressed at 0-30min after
egg-lay (Pilot et al. 2006) are here: Pilot\_0-30min\_Affymetrix\_call.txt.  


---

  
TF expression information for the adult fly was taken from FlyAtlas. Clustered expression data
for dichotomised presence/absence calls (>=3 present calls across 4
replicates = "expressed") is here: adult\_TFs\_present\_call\_discr.txt,
adult\_TFs\_present\_call\_discr.xml,
and
the clustering based on up/down calls in respect to whole-fly
material is here: adult\_TFs\_updown\_discr.txt, adult\_TFs\_updown\_discr.xml.
Both
datasets can be opened in Genesis
to view/extract the identify of TFs in the various clusters.  

## Combinatorial use of TFs

Possible TF interactions (on the basis of TF co-expression) can be
found here: possible\_interactions.txt,
possible\_interactions.xml.
Loading
both files together into Genesis
will reproduce Figure 4B.  
