# Supplementary material for: The developmental expression dynamics of Drosophila melanogaster transcription factors
Source: Genome Biol. 2010 Apr 12;11(4):R40. doi: 10.1186/gb-2010-11-4-r40 (PMC2884543; doi:10.1186/gb-2010-11-4-r40)
Supplement: Additional file 2 — Mini-website: raw data and intermediate results. A self-contained website to browse and retrieve all primary data used in this study, as well as intermediate results such as clustering results. [file gb-2010-11-4-r40-S2.ZIP › raw data/TFs from Table1.html]

TFs from Table1


# TF groups as defined in Table 1

  
BDGP stage 1, tissue Mat  
BDGP stage 1, tissue PoleCell  
BDGP stage 2, tissue AntEnd  
BDGP stage 2, tissue gap  
BDGP stage 2, tissue Segmental  
BDGP stage 2, tissue EctAISN  
BDGP stage 2, tissue FoGut  
BDGP stage 2, tissue DorsEct  
BDGP stage 2, tissue Mat  
BDGP stage 2, tissue Amnio  
BDGP stage 2, tissue VentEct  
BDGP stage 2, tissue Yolk  
BDGP stage 2, tissue Meso  
BDGP stage 2, tissue Phar  
BDGP stage 2, tissue PoleCell  
BDGP stage 2, tissue PostEnd  
BDGP stage 2, tissue ProcephEct  
BDGP stage 2, tissue VisualPr  
BDGP stage 2, tissue Ubiq  
BDGP stage 2, tissue HiGut  
BDGP stage 2, tissue Midline  
BDGP stage 3, tissue AntEnd  
BDGP stage 3, tissue HeadMeso  
BDGP stage 3, tissue gap  
BDGP stage 3, tissue TrunkMeso  
BDGP stage 3, tissue Segmental  
BDGP stage 3, tissue Clypeo  
BDGP stage 3, tissue FoGut  
BDGP stage 3, tissue DorsEct  
BDGP stage 3, tissue VentEct  
BDGP stage 3, tissue Amnio  
BDGP stage 3, tissue VentCord  
BDGP stage 3, tissue Yolk  
BDGP stage 3, tissue Phar  
BDGP stage 3, tissue PoleCell  
BDGP stage 3, tissue PostEnd  
BDGP stage 3, tissue ProcephEct  
BDGP stage 3, tissue VisualPr  
BDGP stage 3, tissue Ubiq  
BDGP stage 3, tissue HiGut  
BDGP stage 3, tissue Midline  
BDGP stage 4, tissue CentBrain  
BDGP stage 4, tissue AntEnd  
BDGP stage 4, tissue HeadMeso  
BDGP stage 4, tissue TrunkMeso  
BDGP stage 4, tissue Segmental  
BDGP stage 4, tissue Clypeo  
BDGP stage 4, tissue FoGut  
BDGP stage 4, tissue DorsEct  
BDGP stage 4, tissue VentEct  
BDGP stage 4, tissue Amnio  
BDGP stage 4, tissue VentCord  
BDGP stage 4, tissue Yolk  
BDGP stage 4, tissue Phar  
BDGP stage 4, tissue PostEnd  
BDGP stage 4, tissue ProcephEct  
BDGP stage 4, tissue Ubiq  
BDGP stage 4, tissue HiGut  
BDGP stage 4, tissue Plasmat  
BDGP stage 4, tissue Midline  
BDGP stage 4, tissue GermCell  
BDGP stage 5, tissue PostSpir  
BDGP stage 5, tissue CentBrain  
BDGP stage 5, tissue Trach  
BDGP stage 5, tissue AnalPad  
BDGP stage 5, tissue FB  
BDGP stage 5, tissue MalTub  
BDGP stage 5, tissue TrunkMeso  
BDGP stage 5, tissue PNS\_Mechano  
BDGP stage 5, tissue HeadViscMusc  
BDGP stage 5, tissue SalGl  
BDGP stage 5, tissue HeadSomMusc  
BDGP stage 5, tissue SNS  
BDGP stage 5, tissue HeadEpi  
BDGP stage 5, tissue TrunkViscMusc  
BDGP stage 5, tissue Yolk  
BDGP stage 5, tissue TrunkSomMusc  
BDGP stage 5, tissue DorsEctEpi  
BDGP stage 5, tissue Ubiq  
BDGP stage 5, tissue PNS\_Chemo  
BDGP stage 5, tissue GermCell  
BDGP stage 5, tissue HeadMeso  
BDGP stage 5, tissue VentEctEpi  
BDGP stage 5, tissue PostMidGut  
BDGP stage 5, tissue Garland  
BDGP stage 5, tissue FoGut  
BDGP stage 5, tissue Clypeo  
BDGP stage 5, tissue CardMeso  
BDGP stage 5, tissue VentCord  
BDGP stage 5, tissue Amnio  
BDGP stage 5, tissue AntMidGut  
BDGP stage 5, tissue Crystal  
BDGP stage 5, tissue OpLobe  
BDGP stage 5, tissue Phar  
BDGP stage 5, tissue HiGut  
BDGP stage 5, tissue Plasmat  
BDGP stage 5, tissue atrium  
BDGP stage 5, tissue Midline  
BDGP stage 5, tissue Proceph  
BDGP stage 6, tissue PostSpir  
BDGP stage 6, tissue CentBrain  
BDGP stage 6, tissue Trach  
BDGP stage 6, tissue
13-16.RingGland  
BDGP stage 6, tissue Gonad  
BDGP stage 6, tissue AnalPad  
BDGP stage 6, tissue FB  
BDGP stage 6, tissue MidGut  
BDGP stage 6, tissue MalTub  
BDGP stage 6, tissue HeadViscMusc  
BDGP stage 6, tissue SalGl  
BDGP stage 6, tissue AdultImagPr  
BDGP stage 6, tissue HeadSomMusc  
BDGP stage 6, tissue SNS  
BDGP stage 6, tissue HeadEpi  
BDGP stage 6, tissue TrunkViscMusc  
BDGP stage 6, tissue Yolk  
BDGP stage 6, tissue TrunkSomMusc  
BDGP stage 6, tissue DorsEctEpi  
BDGP stage 6, tissue Ubiq  
BDGP stage 6, tissue PNS\_Chemo  
BDGP stage 6, tissue Oenocyte  
BDGP stage 6, tissue GermCell  
BDGP stage 6, tissue CardioVasc  
BDGP stage 6, tissue VentEctEpi  
BDGP stage 6, tissue PNS\_mechano  
BDGP stage 6, tissue Garland  
BDGP stage 6, tissue Clypeo  
BDGP stage 6, tissue FoGut  
BDGP stage 6, tissue VentCord  
BDGP stage 6, tissue Amnio  
BDGP stage 6, tissue Crystal  
BDGP stage 6, tissue OpLobe  
BDGP stage 6, tissue Phar  
BDGP stage 6, tissue HiGut  
BDGP stage 6, tissue Atrium  
BDGP stage 6, tissue Plasmat  
BDGP stage 6, tissue Midline  
  
