# Supplementary material for: The developmental expression dynamics of Drosophila melanogaster transcription factors
Source: Genome Biol. 2010 Apr 12;11(4):R40. doi: 10.1186/gb-2010-11-4-r40 (PMC2884543; doi:10.1186/gb-2010-11-4-r40)
Supplement: Additional file 3 — More details on expression classes a and d. A PDF file containing more details on expression classes a and d. [file gb-2010-11-4-r40-S3.PDF]

Characterisation of expression classes A and D.

When we analysed the biological role of these maternal TFs, we were surprised to find many that are putatively involved in organ system development. For example, 73 of the 219 TFs annotated as maternal contribution later show expression in the ventral nerve cord and 69 in the embryonic brain and their respective primordia. Apart from expected functions in regionalisation of the early embryo, these genes indeed show enrichment for GO functional categories in later developmental processes, such as nervous system development, neurogenesis or salivary gland development (all $p < 0.005$). About 20% of the maternal TFs cannot be detected in stages 4-6 and may represent targets of zygotic degradation. These genes do not show enrichment for the previously mentioned GO categories.

A second large group of site-specific TFs is present throughout embryonic development. This set comprises 30.3% (113) TFs in the BDGP dataset, covering embryonic stages 1-16, representing development between 0-16h AEL. These factors show enrichment in the foregut anlage, and both the ventral nerve cord and brain primordia. Interestingly, three quarters of these TFs are expressed in the adult brain (according to FlyAtlas). Taken together with the results on maternal expression, this suggests that a considerable proportion of the TF repertoire is involved in the development and maintenance of nervous system structures.
